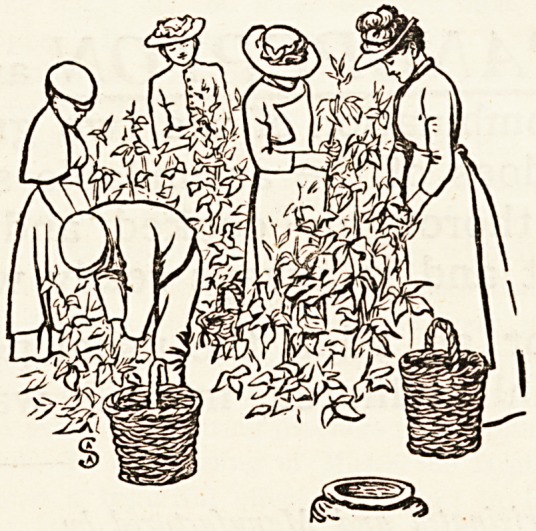# "The Hospital" Nursing Mirror

**Published:** 1900-12-08

**Authors:** 


"The Hospital" Nursing Mirror.

Being the Nursing Section of "The Hospital."

[Contributions for this Section of "The Hospital" should be addressed to the Editor, "The Hospital" Nursing Mirror, 28 & 29 Southampton Street, Strand, London, W.C.]

### Notes on News from the Nursing World.

### THE CZARINA AS A NURSE.

The British as well as the Russian people are rejoiced to know that the Czar is making steady progress, and continues to gain strength. But we should be very sorry if the example he has set in having no nurse but his wife were followed by persons of rank elsewhere. The womanly devotion of the Czarina can only call for the warmest admiration. But it is admitted that she "is somewhat thinner owing to want of sleep and anxiety," which shows that the strain has been greater than she should have been called upon to bear. Further than this, typhoid is one of the diseases which most urgently requires skilled nursing. Nothing could be more disastrous than to encourage the idea that any amateur, however unremitting her care and attention, is able, without serious risk, to nurse a patient suffering from typhoid; and we take the earliest opportunity of expressing our regret that the Czar's "only nurse" has been his wife.

### NURSES ON HORSEBACK.

It is proposed by Mr. A. G. Hales, one of the war correspondents who have lately returned from South Africa, that Army nurses should be taught to ride until they are expert horsewomen. Mr. Hales says: "It seems to me that most of the modern fighting will be done by men who move rapidly in the saddle, and I cannot see why nurses should not accompany them. Each division should possess its own corps of nurses, who could take the field as rapidly as medical men do now." There can be no objection to nurses learning to ride, if they have the wish and the opportunity. The accomplishment may, at any time, prove useful, but the idea of making it a *sine qua non* for admission to the Army Nursing Service will not, we hope, be ever entertained for a moment. There are many women who while splendid nurses would never be at home in the saddle.

### A COMPLAINT FROM NEW ZEALAND.

Miss Emily Nicol, Secretary of the Red Cross Brigade, Auckland, New Zealand, makes a sweeping charge against the military authorities in London. She accuses them of refusing to employ colonial nurses in the nursing of sick and wounded soldiers in South Africa. Now this is really unfair, for our own columns have shown that the Government availed themselves of the services of a considerable number of colonial nurses. Miss Nicol, no doubt, means that applications from New Zealand nurses were declined. It is a pity if, as New Zealand soldiers fought and are still fighting side by side with soldiers from the old country, the offers of New Zealand to send nurses was ignored; but even such a mistake in policy and such a breach of courtesy, would not justify Miss Nicol's assertion that "our men were left to die for want of attention." This is one of the questions which must have come before the Hospitals Commission, and it will be well to await the issue of their report before founding any conclusions on the unsupported statements of Miss Nicol.

### MISS HIBBARD AT HOME.

At the first annual meeting of the Spanish-American War Nurses in New York Miss M. E. Hibbard gave an interesting account of the experiences of herself and the five nurses who accompanied her to England for work on the hospital ship *Maine*. She mentioned that they "had been presented to Her Majesty the Queen, who had addressed to them an appreciative remark regarding their care for 'my men.'" Miss Hibbard spoke in highest praise of the British Army nursing sisters whom she had met in South Africa.

### THE LIFE OF A HOSPITAL NURSE.

A copy of the new number of the *Humanitarian* has courteously been forwarded to us, with an article by Miss Elizabeth French on "The Life of a Hospital Nurse" underlined. This is the second time Miss French has written on the subject, and as she calls her contribution "a rejoinder" to a short note in The Hospital Nursing Mirror of September 15th, we do not like to pass it by entirely without comment. We observe that Miss French now changes her ground and extends her indictment to workhouse infirmaries, supporting her new allegations by two quotations from letters in our own columns in the issue of September 15th. But if she read our pages regularly she would know that we are always ready to afford publicity to the grievances of nurses, and if they are substantial, to endeavour to secure their removal; while as to workhouse infirmaries, the further improvement of nursing in these institutions is one of the objects we have set ourselves to accomplish. As to hospitals, however, it is useless to argue with a lady who says that "there may be exceptional institutions" in which nurses are properly fed and adequately cared for, but that "such places ought to bear some distinguishing mark, such as a flag, showing that here, at least, nurses might expect humane treatment." The hopeless prejudice, or crass ignorance, of a critic who writes in such terms as this, puts her out of court as a witness worthy of any further attention.

### CHRISTMAS AT THE LONDON TEMPERANCE HOSPITAL.

The matron of the London Temperance Hospital says she always reckons that she works harder on the evening of Christmas Day than any other evening during the year; but she derives keen satisfaction from her labours. Though there will be no general decoration of the wards at the popular institution in the Hampstead Road, a large empty ward will be set apart for the purpose. The patients will be allowed to receive one visitor at four, and two at six. The children, for whom a Punch and Judy show is to be provided, will be entertained from six to seven, and the grown-up patients and their friends from six to nine. The entertainment for the latter will include a concert. During Christmas week, every patient, every nurse, and every servant, will receive a parcel from a large Christmas tree. There will be an entertainment for the nurses the first week in the New Year, and the attractions on the occasion are provided by themselves and their friends. On that evening nurses from the Hostel will relieve the nurses on the staff of the hospital, so that all the latter, who would otherwise have been on duty, will be able to attend. The nurses decorate the wards a good deal with plants and flowers. The cost of the whole of the Christmas entertainments for patients and nurses is defrayed out of a special fund collected by the matron for that purpose.

### CHRISTMAS AT GUY'S.

THE matron of Guy's Hospital informs us that the wards will be decorated, "but in a simpler and less expensive way" than formerly. There will be entertainments for the patients as usual, and the Christmas tree which is always placed in one of the wards will be in "Martha" Ward this year. The Christmas dinners for the patients will be just the same as in former years. There will be plenty of good things on the tables, and the men will be allowed to smoke.

### CHRISTMAS AT ST. THOMAS'S.

ENQUIRIES at St. Thomas's Hospital elicited the information that little will be done in the way of Christmas festivities, and that there will be no decorations. Chinese lanterns in the wards is about the sum of what is possible in such a huge institution. The entertainments will not be of an elaborate character.

### CHRISTMAS AT THE CHILDRENS' HOSPITALS.

AT the Hospital for Sick Children in Great Ormond Street the Christmas preparations are not very forward yet, but the matron was able to say that there would be quite as much done for the little patients as usual—Christmas trees with presents, wards lighted with fairy lamps, plenty of flowers and plants, will be the order of the day or rather the week, for everything will take place in the week following Christmas Day. "The wards are planned with a view to sanitation," the matron said, "and we shall not have hangings nor anything that would catch the dust." At the Alexandra Hospital for Children with Diseases of the Hip much the same programme will be followed. The nurses are getting up little entertainments in the different wards, which are already so prettily designed and coloured that much decoration is not required, though there will be plenty of flowers and plants We greatly regret to learn that the matron, Miss Moore, is dangerously ill.

### CHRISTMAS AT THE HOMŒOPATHIC HOSPITAL.

AT the Homœopathic Hospital everyone gets a present except the matron, she laughingly told our representative—"children, out-patients as well as in-patients, nurses, wardmaids, porters—in fact, none of the staff is forgotten." The Christmas tree is lighted with electric light, and the wards look very pretty, with plenty of plants and flowers. The presents for the patients are mostly of a useful description, and take the form of warm clothing. A great deal of preparation is required, for at all times the clothing is provided, and at Christmas it means extra work and thought to choose the right present for the hundred patients inside the hospital, and the many others outside.

### THE CROYDON GUARDIANS AND THE INFIRMARY MATRON.

THE extraordinary action of the Croydon Board of Guardians in reference to the matron continues to attract much attention in the nursing world. From a number of letters we have received we have chosen two for publication this week, and should have selected more, but our correspondents on this subject seem to forget that the space we can devote to one topic, however interesting, is limited. Both the writers whose communications we print have the courage of their convictions and do not conceal their identity. Mr. Berry, while not expressing any approval of the course pursued by the Croydon Guardians, thinks it necessary to contend that no individual matron should be allowed "to be such an autocrat as to have the withholding of a certificate to one she has allowed to go through her training." But a matron must, in a sense, be an autocrat. If she cannot be trusted to use her autocratic power with judgment, she is in a position she is not qualified to fill. The letter of the matron of St. Mary's Hospital, Tenbury, is a specimen of many others, and there is no doubt whatever that Miss Julian has the almost undivided sympathy of the nursing profession, from probationers upwards. Apart from the profession a leading local paper insists that the legitimate outcome of the dispute will be the re-instatement of the matron in her old duties by the Local Government Board. Out of evil good will probably come in this, as in many other cases, and if the crisis at Croydon gives an impetus to the movement for bringing the duties of the infirmary matron up to date, and her position to a much higher standard, there will be some cause for thankfulness that it has arisen.

### DEATH OF A FRIEND OF NURSES.

THE death of Mr. Charles Langton, of Liverpool, has naturally provoked the keenest expressions of regret in the nursing world. Both the District Nursing Association, of which he was vice-president, and the Liverpool Training School and Home for Nurses, of which he was chairman, have passed resolutions deploring his death and acknowledging the services he rendered in their behalf. Mr. William Rathbone, in a letter to the chairman of the Nursing Association, wrote:

> For forty years—indeed, from the very initiation of the work which this association is now carrying on—we have owed it to his tact, influence, and unwearied care that throughout the nineteen districts of Liverpool ladies were always found to undertake the very responsible position, both as to funds and work, of superintending those districts. He only gave up this part of the work in 1898, when on the formation of this association he resigned the chairmanship of the District Nursing Branch. Through all that long period he and I worked together as colleagues, and probably no one has known so long and intimately as I have done what labour, thought, and care this work has entailed upon him, or how much the widespread success of the system of nursing the poor in their own homes is owing to his thoroughness and devotion.

No testimony could be more valuable than that of Mr. Rathbone, to whom nurses will for ever remain indebted; but it may be added that Mr. Langton took a leading part in the management of the Liverpool Training School from the commencement, when it comprised the triple work of supplying the necessary staff of the Royal Infirmary, of conducting the work of district nursing in Liverpool, and of supplying a staff of nurses for the nursing of the sick in private homes.

### ARMAGH GUARDIANS AND THE IRISH LOCAL GOVERNMENT BOARD.

FORTIFIED by the opinion of The Macdermot, Q.C., the Armagh Board of Guardians have determined to defy the

Irish Local Government Board. The Guardians appointed a probationer nurse in the infirmary, who had only been on probation three months, to the position of assistant nurse, whereupon the Local Government Board declined to sanction the appointment, and insisted that a properly trained nurse should be chosen. The master of the workhouse and the medical officer, carrying out the order of the Board, obtained the services of a nurse from Dublin in the infirmary, and the Armagh Guardians, having referred the question of their legal responsibility to The Macdermot, now, by his advice, refuse to pay her salary. The question is one of great importance both to the public and to nurses, and if it is really the case that the Local Government Board do not possess the power to compel the Armagh Guardians to pay the salary of a trained assistant nurse, Parliament must be asked forthwith to give it them.

### A NEW AMERICAN PERIODICAL FOR NURSES.

We cordially congratulate the conductors of the new American periodical for nurses upon the appearance and contents of *The American Journal of Nursing*, published by J. B. Lippincott's Company, 624 Chestnut Street, Philadelphia, as the organ of the Associated Alumnæ of Trained Nurses of the United States. The November issue contains a number of very interesting contributions, including the address of the President of the annual Convention and an article on "Our Floating Hospitals." Under the heading of "Practical Points on Private Nursing," Miss Isabel McIsaac gives some useful diet lists for private duty nurses; there is a section devoted to Construction, Sanitation, and Hygiene; another to Education; a third to Prophylactics, and a fourth to Hospitals and Training-school Items. Miss Lavinia L. Dock is in charge of foreign news. In connection with this department the editor will find, we think, that a neutral and wide-minded treatment of nursing questions, whether at home or abroad, will promote the popularity of the enterprise, which certainly deserves to succeed. It is admirably got up and excellently printed.

### A MODEL HOME FOR NURSES AT TORONTO.

The new nurses' home which has just been opened at Toronto in connection with the General Hospital, is of a very up-to-date character. The apartments of the lady superintendent consist of an office, a bedroom, dining-room, drawing-room, pantry, and bath-room. In the basement is a spacious, well-lighted dining-room for the nurses, with plenty of air and sunlight. Here are also a lecture-room and a bicycle-room. On the first floor there is a handsome library, furnished by a lady in gratitude for the tender nursing which her son received in the hospital three years ago. There are two large sitting-rooms for the nurses, and the rest of the floor, with the two floors above contain the nurses' bedrooms, which are all finished with hardwood floors and are comfortably furnished. Each of the seventy-five nurses attached to the hospital has added many dainty and pretty personal belongings, which give the rooms a homelike appearance; and the home altogether seems to be quite of a model character, affording the best possible evidence of the high appreciation in which members of the nursing profession are held in the Dominion.

### "LADIES OF THE RED ROBE."

The nurses of the Bradford District Nursing Association, who are known in the poor parts of that town as the "Ladies of the Red Robe," on account of their conspicuous uniform, have just removed to more commodious headquarters in Horton Lane. The new premises are much better adapted for the requirements of the nurses, of whom there are nine, in addition to the matron. The funds for the work are mainly raised as follows:—A lady, who is interested in charitable work, is asked to become president of a district, or circle, and, should she accept, she undertakes the responsibility of finding a number of the vice-presidents to act under her. The latter, in their turn, obtain a number of associate members, and each lady offers a small subscription. There are a large number of these circles, and last year the income from this source amounted to about £400. It need hardly be added, however, that there is always plenty of room for more "circles."

### THE LONDON SCHOOL NURSES' SOCIETY.

The Countess of Aberdeen appeals for help on behalf of the London School Nurses' Society, which we are sorry to hear is in such dire financial straits that the chairman and committee are compelled to consider the advisability of relinquising its work altogether. It will be recollected that the organisation was only established two years ago by Miss Honnor Morten with the sanction of the London School Board; but, as Lady Aberdeen points out, the results of these two years are amply sufficient to show what a boon the visits of these nurses are to the children in the poorest localities. There are five nurses, each with ten schools under her care, every one of which she visits twice weekly. As our readers know, the Queen Victoria Jubilee Nursing Institute has inspected the work of the nurses and has pronounced it to be satisfactory. In fact, it was hoped that the income would soon be increased so as to allow of the employment of eight nurses. Instead, it has become a question of entirely abandoning the movement at the end of the year. We hope that the response to the President's appeal will at any rate be such as to ensure the continuance of the Society, with prospects of the much-needed extension of its efforts.

### KIDDERMINSTER INFIRMARY.

The annual examination of the nurses at the Kidderminster Infirmary took place last week, and the results were very satisfactory, both to the examiners and to the lecturers. Three nurses qualified for certificates, Nurse Ethel Cocks gaining the President's prize of a book; Nurse Eastwood came out first on the list of the Junior Probationers. The lectures are given by the matron and the house surgeon. The examiners are four members of the hon. medical staff—Mr. Lionel Stretton and Mr. Hodgson Moore for the senior nurses, Dr. Penrhyn Evans and Dr. Bertram Addenbrooke for the juniors.

### A ROMANCE OF THE SPANISH WAR.

The marriage of Miss Harriet Gaddis to Lieutenant Lee in Havana is announced. The bride was a graduate of the Garfield Training School for Nurses in Washington, and was sent by the United States Army Department to assist in nursing the soldiers during the Cuban War. Lieutenant Lee was one of her patients, and she nursed him back to life.

### Lectures on Nursing for Probationers.

By E. MacDowel Cosgrave, M.D., Lecturer to the Dublin Metropolitan Technical School for Nurses.

### No. XX.—DIET FOR THE SICK.

It often happens that only general directions as to diet are given by the doctor, and it is left to the nurse to fill in the details.

A few general hints should be kept in mind:—Do not ask the patient "What will you have?" or tell beforehand what food is to be given; the element of surprise goes a long way in the monotony of illness and convalescence, and thinking of food beforehand does not promote appetite. Let the food be well-cooked and served punctually. Let the cloth be clean, the silver bright, and omit nothing. Do not bring too much. Have the patient ready, comfortably propped up and warmly covered, but with the arms free. When the meal is over clear away. Study the patient's likes and dislikes, and try by tact to overcome fads and whims. In illness a more liquid diet is required than in health; the digestive glands often do not secrete so well and absorption is not so active. This is especially true of fevers, and, indeed, of all illnesses with raised temperature, where not only are solids badly digested, but the increased burning leaves more waste to be washed away, and for this purpose more liquid is required.

Milk is called a perfect food, as it contains all the classes of food in right proportion; it is the natural food of the infant, and is quite sufficient for the first few months of life. If milk be let stand the cream gradually rises to the top; this is often removed, and the milk sold as pure although it has been robbed of one of its most valuable constituents. The richness of milk in cream can be measured by pouring milk into a tall glass tube graduated into 100 parts. After standing for twelve hours the cream should occupy 8 to 10 or even more parts. If there is less the milk has either been skimmed or watered. If milk is kept too long it sours—that is lactic acid forms and throws down the curd. To prevent souring boracic and salicylic acids are added, but their addition is injurious. Bread-soda and a little sugar will also postpone souring. If milk is even commencing to sour it is dangerous, as the heat of the digestive canal increases the rapidity of the souring, and so intestinal irritation and severe diarrhœa may result from milk which is only slightly tainted. When infants have to be bottle-fed it is well to remember that human milk and cow's milk have not the same properties. The composition of average samples is shown in the following table:—

| | Human. | Cow's. |
|---|---|---|
| Caseine ... ... ... | 2 | 4 |
| Fat... ... ... ... | 4½ | 4 |
| Milk sugar ... ... ... | 7 | 5 |
| Salts ... ... ... | ½ | 1 |
| Water ... ... ... | 86 | 86 |

The easiest way to make cow's milk like human milk is as follows:—To a pint of cow's milk add the cream and whey of another half pint, separated by sweet essence of rennet, in which an ounce of milk sugar has been dissolved. Milk sugar should always be used for sweetening milk for infants.

Another disadvantage of cow's milk for infants is that in the process of digestion it forms large and tough curds; on the other hand human milk forms small soft curds, not larger than rice grains. It is these curds which so often cause indigestion in children fed on cow's milk; curiously enough, condensed milk and dried milk form smaller and more digestible curds than fresh cow's milk. In digestion, the rennin in the gastric juice causes curds to form, as this is the first step in the digestion of caseine.

There are a great many methods of preventing large curds forming. The addition of sodawater, limewater, or barley-water mechanically prevents the newly-formed curd running into large masses. The addition of a little water with a lump of sugar dissolved in it acts to some degree. Boiling milk checks the formation of large curds, but renders the curd harder to digest.

If these methods fail, the milk should be partly or wholly peptonised; peptogenic milk powder or peptonising powders may be used; when the peptonising has been carried on far enough the milk should be brought to the boil to stop the process, which if carried too far would make the milk unpleasant.

For older children or grown-up persons a little powdered biscuit or carbonate of magnesia shaken up in the milk will act mechanically. The milk may be curdled by the addition of rennet, the large soft curd being afterwards broken up with a fork. Hot milk is more easily digested than cold; and milk brought to the boil and cooled to drinking temperature with soda-water is often retained, even if there is nausea. The different parts of milk may be given separately. Cream is a good heat-giving food, and checks wasting; it is concentrated, and so is easier digested if added to milk. Whey, which is rich in salts, is a good diuretic. Cheese contains all the proteids and much of the fat; it is, however, too concentrated to be easily digested. Butter contains all the fat and some of the proteids, together with added salt.

Milk is very often the vehicle of infection. Scarlatina and typhoid epidemics have in several instances been traced to the milk supply, and the bacillus of consumption is frequently present. To avoid these dangers the milk should be sterilised—that is, heated to at least 185° Fahr., at which temperature all contained germs are killed, and kept from fresh infection.

Plenty of good sterilisers are on the market, and are used in most large institutions. The easiest way to sterilise a small quantity of milk is by placing it in the centre compartment of a double saucepan with cold water outside; the latter should be brought to the boil, and allowed to boil for five minutes, then without removing the lid the inner saucepan should be lifted out and placed in cold water, so as rapidly to cool its contents.

Beef-tea, although it does not contain a large quantity of flesh-forming proteids, is a grateful and useful food in case of illness. If kept well below boiling point during making, its food value is greatly increased, as its albumen is not hardened into scum. Chicken broth contains a little more nourishment, and often agrees better than beef-tea. Mutton broth and veal broth are useful for variety. Sometimes the particles in beef-tea irritate a sore throat; this can be lessened by pouring the hot beef-tea on some pearl barley, and allowing it to "draw" until cool enough to drink. Many bought preparations—whatever their stimulant value—have practically no food value. The preparations in jelly form and many of the fluid form are of little value. Amongst the really useful are Bovril, Bovinine, and Beef Peptonoids; the latter has 10 per cent. of useful proteids.

Eggs, like cheese, are too concentrated to be easily digested; this can be remedied by beating up with water and adding a little salt. Eggs are better poached or boiled than when fried. Boiled eggs are most digestible when lightly done, or when hard boiled for a considerable time.

Starchy foods can be predigested by the addition of pancreatic fluid; this must not be added until the food is nearly cool enough to eat, as greater heat kills the ferment. Malt extract can be used for the same purpose in the same way.

(*To be continued.*)

### Examination Questions for Nurses.

### PLAGUE NURSING.

### RESULT OF NOVEMBER COMPETITION.

THE question was as follows:—"State what you know of the best means of nursing plague, what steps you would take to avoid the spread of infection, and what measures you would adopt to insure personal immunity whilst ministering among the sick."

### THE FIRST PRIZE.

Nurse Eilrah takes the first prize. She mentions the danger arising from the presence of rats. Undoubtedly they convey infection, though the exact mode is not known.

### THE SECOND PRIZE.

Nurse Hancox is the second successful competitor. Her answer is very good, but she speaks of "going among outsiders," which would certainly not be permissible while nursing plague. This lady almost invariably sends good, practical, well-thought-out answers, showing a thorough interest in her work, but also, almost equally, invariably, through some oversight (perhaps but a small one), fails to come out first. Her work is so good that, with a little more care and thought, it would be first class. Both these successful candidates have noticed the need of keeping under observation those who have been in contact with the sick in the early stages of the disease and before it has been developed.

### HONOURABLE MENTION.

Only one honourable mention is awarded, and Nurse Adèle gains it.

### ANSWER TO QUESTION FOR NOVEMBER, 1900.

### FIRST PRIZE.

As in all infectious cases, it must be notified to the public officer of health. The patient must be isolated; the room must have as little furniture as possible; there must be extreme cleanliness and plenty of ventilation. There are several types of plague, viz., the pneumonic, bubonic, septicæmic, and enteric, &c., the pneumonic being the most dangerous and infectious. The symptoms are: rigors, ivomiting, headache, furred tongue, high temperature (though in some cases not above 100°), extreme prostration and ethargy, and sometimes delirium; generally there are swollen glands, forming buboes, in the groin, axilla, or neck: these are very tender and painful; they may absorb or suppurate, or have to be opened; the discharge is very offensive and infective. The skin, bowels, and kidneys are inactive.

The patient must have a light nourishing diet; he must be kept in a recumbent position, and no sudden movement should be allowed, as death is often due to heart failure. Great relief is given by heat applied in some form, such as fomentations or poultices to the buboes, but of course all treatment will be ordered by the doctor. Vapour baths may have to be given.

To avoid the spread of infection the usual precautions in infectious work should be taken, viz., disinfection of excreta, urine, and sputum, with some disinfectant, such as 1–500 perchloride of mercury, all bed and body linen being steeped in the same fomentations and poultices, &c., should be burnt, and the patient should only use a piece of rag as a pocket handkerchief, which should be burnt immediately after use. All flies, gnats, or fleas (if found in the patient's room) should be destroyed, as they may spread infection. There should be *thorough* fumigation of the patient's home, and all persons who have been in contact with him from his first feeling ill should be isolated and kept under observation for twelve days, the incubation being from two to twelve days. All rats in the house and neighbourhood should be caught and killed, as they are susceptible to plague and are a great means of spreading it.

Before undertaking to nurse plague a nurse should be inoculated with Haffkine's Fluid or Yersin's Serum. All the ways by which the infection of plague is communicated are not known; but certainly through the nose or by a wound, so the nurse must be most careful to avoid having (or to cover) any scratch or cut, and she should endeavour not to inhale the patient's breath, especially in the pneumonic case. She should disinfect her hands after touching the patient or anything used by him, and be sure to use forceps for all dirty dressings. She should wear an overall while in the patient's room, taking it off on going off duty.

EILRAH.

### SECOND PRIZE.

Immediately remove patient from her surroundings and isolate. She must be kept absolutely clean, old body linen to be used if possible, that it may be destroyed as soon as removed. A plentiful supply of good nourishing diet to be given. The room to have a fire burning, and to be well ventilated. Eyes, nose, and mouth to be cleansed frequently with some disinfectant lotion, and the swabs at once burnt. Glands and buboes will probably be dressed with soothing lotion dressings, and great care must be taken with regard to soiled dressings, discharges, sputum, &c., that they are burnt immediately or removed. Forceps are to be used, and not nurse's fingers.

The room to be washed over at least twice daily with some disinfectant; all utensils to be kept absolutely for the patient's use alone, and thoroughly cleansed after use, with hydrarg. perchloride 1–1000 or carbolic lotion 1–20.

To prevent the spread of disease, all occupants of the house to be placed under observation for at least sixteen days from the date of removal of patient, and the house submitted to a thorough fumigation. Cats and dogs which have been in contact with the disease must be destroyed. All drains, sinks, &c., to be well flushed with water several times a day, using disinfectants at intervals. All excretion must be covered with perchloride of mercury 1–1000 before being emptied down the water closet. With regard to nurse's health, she must observe the strictest rules and minutest details concerning hygiene. Bath daily, plenty of nourishing food, open-air exercise, keep the bowels open. Take care of small cracks and scratches on hands. Be particular to use forceps when touching soiled dressings, rags, &c. She must wear an overall and cap while in patient's room, and be careful to change her clothing before going amongst outsiders.

NURSE HANCOX.

The chief faults discernible in this month's papers are two in number. First, almost all competitors give elaborate details as to the usual precautions taken whilst nursing in fectious diseases. These should have been passed over merely with a statement that all such precautions would have been taken, then the writer should have emphasised the further efforts that must be made in nursing plague (caused by the peculiar and as yet but imperfectly understood modes of conveying the disease) such as the destruction of animal and insect life. One point considered to be very important by those best calculated to instruct us is entirely ignored by all the candidates, and that point is the necessity for closely examining the bare feet of native attendants and friends of the patients. It is considered that infection is conveyed through slight excoriations on the feet, caused by small cuts or the bites of insects; it is therefore very important to examine carefully the extremities of those who walk barefoot in the neighbourhood of the sick.

The second fault I observe in this month's papers is a tendency to dilate learnedly on the symptoms of different developments of plague. This is quite unnecessary; it is the doctor who is required to diagnose cases and to order treatment, and the nurse who must by her attention to his orders and her quickness of observation and resourcefulness alleviate suffering and promote recovery.

There is one more point I should like to notice. Plague is usually of an endemic character, and therefore is seldom met with in isolated cases, whereas most of our writers treat the matter as they would when called to a solitary case of small-pox or scarlet fever. The nursing would be probably in hospitals or hastily-erected clusters of huts—anything but well supplied with ordinary conveniences.

### THE QUESTION FOR DECEMBER.

How would you prepare a bed for a case of fractured thigh at half an hour's notice in a house belonging to a middle-class patient? This is a very simple question; observe that you are not asked *anything* about treatment. Avoid prolixity. One competitor sent in an answer last week of over 800 words. Our rules are few and simple, but they must be obeyed.

### The Nurse in Hot Climates.

By EDWARD HENDERSON, M.D., F.R.C.S. Edin., late Surgeon, General Hospital, Shanghai.

(*Continued from page* 116.)

Uncooked salads should, as a rule, be avoided in the East. When such a disease as cholera is epidemic salad materials bought in the market become positively dangerous. Vegetables should all be washed under the tap before they are cut up on the kitchen tables. The precautions to be taken regarding fruit have been already detailed.

*Personal.*—The nurse should always use running water to wash her hands when running water can be got. If the supply is from a main or cistern she will have no difficulty in doing this, provided the tap is placed, as it should be, at some little distance above the basin it supplies. To wash the hands in a basin in the usual way is to keep them more or less in contact with the dirt which it is the object of the washing to remove, and if the dirt is of a dangerous kind such washing may be insufficient. When the hands have been soiled in attendance on a case of cholera or the like, the nurse should wash them with turpentine before she uses soap, and may then as a further precaution immerse them in some antiseptic solution. The nurse must of course never eat before her hands have been thoroughly washed, and all soiled garments should be got rid of before she sits down to table.

Wounds on the nurse's hands, however trifling, should always receive attention, and must of course never be neglected for a moment when she is in charge of cases such as plague, where the contagium is one which can be inoculated. A convenient and reliable covering for small wounds is a dressing made with friar's balsam and collodion strengthened by a little cotton. A few threads of cotton wool, preferably absorbent cotton, are laid on the cut or abrasion, and friar's balsam is painted over these with a camel's-hair brush, care being taken not to displace the cotton. After a few minutes, when the balsam has dried, collodion is brushed over the whole. If this dressing is properly applied it may be safely trusted, and the hands may be washed without fear of detaching it. The dressing will last for several days, but in case of accidents fresh collodion had better be brushed over it from time to time.

Mention has been made of an antiseptic mouth-wash or gargle, as a prophylactic to be used by the attendants in cases in which infection is believed to enter by the mouth or air passages. The mouth-wash used, and apparently with good effect, by the men engaged in cleaning the Chinese quarters in Hong Kong in 1894 when plague visited that colony, was one in which carbolic acid, spirit of camphor, and Eau de Cologne were combined. Boracic acid is sometimes employed as a mouth-wash, and a saturated solution—the proper strength to use—is easily and quickly prepared by adding the crystals to water at the temperature of the air, in such quantity that after shaking the containing vessel and allowing it to settle, a distinct sediment, the excess of the salt, is seen to be deposited: this has the merits of safety and convenience, but unfortunately such a solution can only be regarded as possessing mildly antiseptic properties.

*Cholera and Plague.*—Besides the precautions already detailed, the disinfection and destruction of fomites are of course matters which must always be attended to by the nurse in charge of infectious cases. The specific bacillus of cholera is quickly destroyed by desiccation, but continues to multiply outside the body in the presence of moisture. On this account all bed-coverings and articles of clothing, &c., which have been soiled by the discharges from a case of cholera must be quickly and thoroughly disinfected or destroyed. The bacillus of cholera is rarely found in the matters vomited, but these should also be disinfected.

In such a highly infectious disease as plague, everything which has been in contact with the patient which can possibly be spared should be burned. In plague the disinfection of the sputum, and the immediate destruction of any dressings applied to discharging surfaces, are matters which need special attention. The disinfection of the bowel discharges should in plague be continued during the convalescence of the patient; the specific bacillus is reported to have been found in the fæces a month after the crisis of the illness had passed.

Protection against cholera and plague by prophylactic injections has already been discussed.

*Malaria.*—If the nurse is at any time specially exposed to the poison of malaria, in addition to the careful use she makes of her mosquito net—a matter already fully detailed and insisted on as regards her patients—she should take a small dose of quinine daily. Three to five grains is a sufficient quantity, and the dose is one which may be continued for a month or two at a time without harm or inconvenience.

Properly regarded the mosquito net is not only a protection for the healthy, but an important means of isolating the sick. For if the mosquito could no longer obtain access to blood in which the parasite of malaria was circulating, its *rôle* in continuing the life of that parasite in its own body, and eventually transmitting it in an altered form to man, would be ended. It would indeed be well if it were possible, that everyone in whose blood the malarial parasite was known to exist could be kept under cover till cured. Patients suffering from malaria should certainly have the full benefit of mosquito nets, both for their own sakes and for the sake of others.

It is very desirable that the nurse who goes abroad should acquire some knowledge of the mosquito and its habits. Anopheles is the name given to that variety of the insect which, so far as we know at present is alone capable of infecting man with the parasite of malaria. If the nurse is able to recognise the Anopheles larvæ, floating as they do horizontally on the surface of stagnant pools, she might, armed with a little Jeyes' fluid or kerosene oil, materially improve the sanitary condition of her immediate surroundings so far as malaria is concerned.

In a malarious district, however, and indeed wherever mosquitoes are at all troublesome, it is advisable that an addition of Jeyes' fluid or kerosene oil be made to all stagnant pools in the neighbourhood of dwelling houses, as a routine practice not necessarily dependent on any special knowledge of the insect or its habits. These additions need not be large: if kerosene be used, the surface of the water should be covered with a thin layer of the oil, and a very small quantity will do this sufficiently. One part of Jeyes' fluid in 10,000 parts of water is reported to kill the larvæ of both Culex and Anopheles in a few hours.

*Small-pox.*—Small-pox in hot climates is a disease which usually makes its appearance during the colder months. In China, where inoculation is still extensively practised among the natives, and the winter is chosen for the performance of the operation, the regular recurrence of small-pox in cold weather is certain. Prophylaxis by isolation where natives are concerned is at present impracticable. In the East the only protection against small-pox on which dependence can be placed is that conferred by efficient vaccination. The early vaccination of infants and the re-vaccination of adults are matters which need greater attention abroad than at home. The personal protection of the nurse, to be secured before she leaves England, has already been discussed.

THE END.

### Echoes from the Outside World.

### AN OPEN LETTER TO A HOSPITAL NURSE.

THE hopes of the ex-President of the Transvaal that the European Powers were likely to help him with arbitration, which had been steadily fed by the enthusiasm of the crowds in France and Belgium, had a sad blow on Monday. The German Emperor informed him in polite language that his numerous engagements would prevent him from meeting Mr. Kruger in Berlin. Therefore the traveller, who had got as far as Cologne, had to turn tail and go on to the Hague. It is even said that he cried when he received the intelligence; anyhow, he cannot fail to have felt that he had practically lost the game. He had risked his trump card, and it had not proved high enough to take the trick. His assertion that he shall go on to St. Petersburg after Holland will probably not be fulfilled. The Czar can plead his recent illness as a reason for refusal, just as the Kaiser pleaded his hunting appointments. The young Queen of Holland, with the natural enthusiasm of youth for the vanquished, may and probably will welcome the disappointed old man, but she is powerless to do more than offer him her sympathy. Mr. Kruger will have ultimately to decide on a suitable resting place to end his days, for, though his "leave of absence" is only for twelve months, his chance of returning to the Transvaal is exceedingly remote. Meanwhile, Lord Roberts has already left Pretoria, and bids goodbye to South Africa next Tuesday. Rumour speaks of £100,000 and a dukedom as the official recognition of his services. Lord Kitchener remains at the seat of war—for alas! conflict has not yet ceased—and will most likely find that nothing but a rule of iron is possible for the double-faced Boers. If so, he will not be slow to carry it out.

THE reform of the public house is a movement in which all nurses are interested, because, as I said a week or two ago, so many of them come in contact with the terrible results of drunkenness. Compulsory total abstinence is one thing, and the improvement in the quality of the liquor sold quite another. The admirably managed hotels and restaurants in the country have no more to fear than the best brewers, wine merchants, and distillers from the organisation of which Earl Grey is the provisional chairman. The movement instituted by the Publichouse Trust Company, which, having justified its existence and made good its footing in the North of England, is now about to be extended to London, is aimed only at the vendors of bad liquor and the publicans whose one object is to get their customers to purchase intoxicants. The disastrous consequences of drinking adulterated beer have just been demonstrated in a striking manner, and there is no doubt that the consumption of adulterated spirits is also physically and morally ruinous. In any case, the sale of intoxicants should not be pushed, and all public-houses should be, in a real sense, refreshment houses and not merely drinking bars. Earl Grey, in taking the lead in a campaign against the publicans who not merely sell, but push the sale of, poisonous alcoholic drinks, will enlist the sympathy, and command the support, of all advocates of true temperance.

EVEN those whose school days are a long way back can probably remember being taught in their early lessons that the Ancient Britons strove to render themselves beautiful by staining their skins with a blue pigment called woad. It is said that history repeats itself, and apparently the period has come for human beings once more to paint themselves for decorative purposes. This time it is not untutored savages, but ladies of fashion who are the walking pictures. You are probably aware that painted silks, satins and velvets have been the favourite material for evening gowns amongst the wealthy for some months. But now the idea threatens to go farther. Trails of flowers are to be painted say across the front of an evening bodice, a stray bunch of blossom or a dainty tendril to be continued on to the skin so as to touch the neck or cluster on to the shoulder. The strap of velvet which does duty for a sleeve has, may be, a spray of ivy and forget-me-nots painted upon it. The same decoration is carried down almost to the bend of the arm upon the fair wearer's flesh, and it is easy to see that the novelty is capable of much expansion. Women who are clever with their fingers and carefully imitative will find congenial occupation in going round to paint ladies dressing for dinner or ball, except when a lady's maid can be instructed to become a successful human artist. As the notion is still young, it is impossible to say what measures are adopted to prevent the colour running in a heated atmosphere, or whether any injury is likely to supervene by constantly applying colours to the skin. Those are details of no interest to ordinary individuals.

ANY lovers of art who revelled in the Romney Exhibition of last spring will naturally bend their steps towards the Grafton Gallery to see what is called the second edition, and will come away delighted that they have not missed an opportunity of making the acquaintance of many works of the great artist which they have never had a chance of gazing on before. The reason of this is because the organiser of the show has been allowed to withdraw temporarily from their places on the walls the valued possessions of many country homes. Of course there are a few old favourites—and we should enjoy the exhibition far less if there were not—but the majority of the canvases are new friends. Of portraits of Lady Hamilton there are naturally a goodly number, beginning with a very early sketch, and ending with the "Head of Lady Hamilton," the last portrait of the "divine lady" painted from life. One of the most beautiful amongst the collection is that of Lady Hamilton attired as a Bacchante leading a goat, whilst a dog is jumping for joy. The picture is possibly well known to most of you, and the life, the gleesomeness, and the beauty of the painting will probably make you, as it made me, glad to see it again. Of other pictures I especially liked the clever treatment of "Mrs. Whateley" and "Serena Reading," not the two profile pictures, but the one taken from the front, where the lady (Miss Honora Sneyd) is leaning over the book in her lap. The softness and the sweetness of the colouring is haunting, and in a mysterious way so impresses itself upon the brain that it can be easily recalled at will.

IT is always pleasant to hear of any movement for the moral improvement of those who have to undergo a term of imprisonment, and to observe that the old-fashioned idea of anything being good enough for a criminal is fast passing away. Because a man or woman has taken one false step it does not necessarily follow that it must be succeeded by another, and anything which helps to soften rather than to harden, to bring out the best part of the culprit's nature, instead of the worst, must be a move in the right direction. Therefore, two new regulations which have lately been made by the prison authorities, though trifling in themselves, are worthy of note. For the future prisoners may have the photographs of their relations hung in their cells; and when taken home by a warder, the officer is to be in civilian clothing, not in uniform. The first regulation especially says "relations." Accordingly, I fear that the woman whose heart has been delivered into the keeping of the man whom she hopes to call husband will still be refused permission to gaze hourly upon the loved features; but no rule can be all-embracing, and it is a great thing that a man may have the picture of his wife or his little ones to help him through the darkest hours. Also that, as far as in them lies, the authorities wish the prison life to be left entirely behind as soon as the prisoner passes over the threshold of what in a broad sense should be "the house of correction."

### Functions and Entertainments.

### ROYAL BRITISH NURSES' ASSOCIATION CONVERSAZIONE.

ABOUT eight o'clock on Tuesday evening the familiar blue bonnet and cloak might have been seen wending its way towards Westminster, where, in the Town Hall, Caxton Street, the Royal British Nurses' Association was holding its annual conversazione.

The guests, in all about 400, were received by the Vice-chairmen of the Association—Miss Thorold, Sir J. Crichton Browne, Mr. Pickering Pick—and the hon. officers—Mrs. Coster, Mr. Langton, and Mr. Fardon—while the Æolian Ladies' Orchestra played in the gallery above, and tea and coffee were served. By a quarter to nine the room was full, and one moved about with difficulty. The uniforms predominated, though some visitors were in evening dress; and the bright scarlet capes of a few Reserve nurses shone out among the quieter colours.

At nine o'clock a general move was made downstairs to the entertainment given by the various ladies and gentlemen who had kindly promised their services; and there was a good deal of disappointment when it was announced that Canon Fleming had been prevented from being present. More than one nurse was heard to say, "I should like to have heard him recite 'Little Emmie.'"

Mr. and Mrs. Stepney Rawson were very effective in their vocal duets, and, in conjunction with Madame Lena Law and Mr. Sterling Mackinlay, were warmly applauded. The amusing song about a crocodile, sung by Mr. Stepney Rawson, was a great success: this extraordinary beast was five miles long, and when he died took ten years to get cold, because he was "so very long and wide." Mr. James Dunn, of the Palace Theatre, seemed to be greatly appreciated in his banjo sketches, judging by the amount of laughter heard all over the room. Mr. Robert Manning was very good in a humorous Irish song, and Madame Lena Law was greatly appreciated. The platform was prettily decorated by the Misses Langton.

In a small room close by, a group of guests might have been seen at any hour during the evening sitting round the electrophone, the instrument at their ears, and a look of concentration on their faces; they were listening to performances at some of the leading theatres in London—the distant orchestra of the Shaftesbury, or the rather noisy performance at one of the music halls, with interruptions from the audience in the gallery.

Altogether, it was a most enjoyable evening, and, as a nurse observed on the stairs, "It was so nice meeting everyone." The refreshments were evidently much appreciated. Among the company were—Miss Young, Matron of Guy's Hospital; the Rev. Dacre and Mrs. Craven; Miss Cattell; Miss Davidson, of Friedenheim; Miss Bowditch, Dr. and Mrs. Griffiths, Miss Robbins, Dr. and Mrs. Hawkins, Mr. Arthur Barker, Dr. Comyns Berkeley; Miss Islip, the Secretary of the new Auxiliary Society; Miss Jackson, Secretary of the Chartered Nurses' Association; and, of course, Miss Leigh, of the Royal British Nurses' Association.

### TABLEAUX VIVANTS AT HAMMERSMITH AND BATTERSEA.

TWO most successful entertainments were given last week in aid of the District Nursing Associations of Hammersmith and Fulham, and South London. The first was in the Town Hall, Hammersmith, and the second in the Battersea Town Hall; and, with the exception of one or two musical items, they were identical.

The first part consisted of tableaux from the Old Testament, and each one was a picture of real artistic merit. The colouring and grouping were in excellent taste, and the figures graceful and pleasing. It is difficult to select, but perhaps the three tableaux of the Shunamite's Son were the strongest in dramatic interest. The little boy among the gleaners, when he said to his father, "My head, my head," was extremely pretty; in his hand was a trailing bunch of large red poppies and ears of wheat, which he still grasped loosely when, in the next scene, he lay on his little bed, and Elisha, clad in green, flowing garments, and with a grand beard, stood with arms upraised. It was difficult to believe that the fine figure on the stage was not that of a real Hebrew prophet. In the third scene the child is sitting up, while his mother is kneeling, with arms stretched out, as the prophet restores her son to her. Another very pretty scene was Ruth Gleaning. In the hot sunshine of the harvest field, the varied colours of the Syrian dresses, and the graceful figures of the gleaners, shading their eyes to watch the meeting with Boaz, were very effective. Other scenes, not less artistic, were of the clashing of cymbals and the playing of long pipes and tabrets, in the Daughter of Jephthah, and the Song of Miriam; and the first musical instrument, where Jubal plays on his shell, with a group of admiring listeners round him, was not less beautiful. In the finding of Moses, the large peacock fans produced a beautiful effect of colour; in Ruth and Naomi, both the principal figures were very graceful, and not less so was Orpah, departing in haste and looking back. Rebecca at the well was another very pretty picture, a group of Eastern women carrying water pots, a child sitting on the well, Rebecca, tall and graceful, with the pitcher, looking down at Eliezer kneeling at her feet offering a necklet.

In the intervals between the tableaux various songs were sung by friends of the associations, the Shepherd's Cradle song being quite one of the sweetest; it was sung at Hammersmith by Miss Gertrude Sickel, and at Battersea by Miss Dillon.

The second part consisted of scenes from the life of the district nurse, and these simple and homely pictures were greatly appreciated by the audience at both performances. First the nurses were seen in the early morning, ready to start on their rounds—one reading her letters, a third making sure that she has everything in her bag, and another inflating her bicycle tyres. Next was the arrival at the house of the chronic patient, where an old be-shawled figure sat in its chair, and the children played on the floor or watched the nurse as she made her daily inquiries of the poor old creature, who hardly seemed conscious of her presence. The Nurse in the Board School was the third picture, and here, backed by a large map of England, nurse was seen putting a roller bandage on a little boy's hand, while one girl laced up the boots of a little tot on the table, and another sat on a bench looking up in the nurse's face. It was amusing to catch a glimpse of the child who did not realise that the curtain was going up, and whose head went rapidly round to the right position; they were all the children of patients in the district, and did their part admirably.

"After the Day's Work" was the last picture, and here the recreational side of the district nurse's life was shown. A group of nurses is sitting together, one is pouring out tea, while others are chatting or reading, and one is singing to the guitar accompaniment of a friend. Of course the parts were acted by friends, not the nurses themselves. It is satisfactory to learn that both entertainments were a great financial success. The halls were crowded, and at Battersea, although there is accommodation for about 1,300 people, applications had been refused. There was only standing room for late-comers at Hammersmith, which made a profit of £40, while at Battersea the net proceeds amounted to £75.

### A Cluster of Fruit Farms.

COULD you but witness the conditions under which Chivers famous Gold Medal Jams and Jellies are prepared, you would not be surprised at their great popularity and high reputation. It is in the pretty Cambridgeshire village of Histon that the process of manufacture takes place. The freshly gathered fruit, chiefly grown upon Messrs. Chivers' own farms (covering many acres of land at Histon, Haslingfield, Haddenham, and Impington), is made into Jam, or the Juice extracted for the flavouring of the Table Jellies. The various processes are carried on in a model Factory, equipped with every modern convenience. Throughout the whole process the most watchful care is exerted to *ensure strict cleanliness and purity.* Pure ripe fruit Juices are employed to flavour the Jellies, and *absolute freshness of material* is always insisted upon. Thus Chivers' Jellies have earned such a reputation for consistent excellence and purity that the public have perfect confidence in them. The *Edinburgh Medical Journal* says: "A perfect form of Table Jelly. The Orange tasted exactly as if a squeeze of fresh orange had been added." A Yorkshire Vicar writes: "Your perfect success will, I trust, remove for the future all those prejudices which people had previously cherished with regard to ready-made jellies. That your great success may reach every sick room as well as hospital ward is my earnest wish." The *Charity Record* says: "A remarkably delicate aroma. We safely predict an increasing popularity as time goes on."

Chivers' Gold Medal Jams and Table Jellies are sold by Grocers and Stores throughout the United Kingdom. *Insist on being supplied with Chivers'.* A free sample packet of Jelly will be sent on receipt of post-card. S. Chivers and Sons, Histon, Cambridge.

### Our Christmas Number.

With our Christmas Number, which will be issued next week, December 15th, will be presented to our readers five handsome plates: "Within the Wards," "Christmas Tree Corner," "A Ward in the Roof," "Delicate Tracery," and "A Corridor." The principal features of the letterpress will be a series of sketches of Christmas at home and Christmas in the hospital. Orders should be given at once to the publisher.

### Presentations.

Camberwell Infirmary.—The nurses of the Camberwell Infirmary have sent a "silver pap bowl" to the infant daughter of the Medical Superintendent, with their sincere good wishes.

Ruchill Hospital, Glasgow.—Miss E. Howe has been presented by several members of the night staff at Ruchill Hospital with a handsome silk umbrella, gold mounted, on the occasion of her leaving that institution to take up private nursing.

Norfolk and Norwich Nurses' Association.—Last week the nurses of the Norfolk and Norwich staff of nurses presented their lady superintendent, Miss Edith Watson, with silver-backed brushes, comb, and scent bottles, as a token of their love and esteem.

Musselburgh District Nursing Association.—Miss Murray, late Queen's Nurse, Musselburgh, has been presented, on her retirement, by the townspeople with a purse of sovereigns and a very handsome gold badge and chain bearing the town arms, and a suitable inscription. The presentation was made in the Council Chambers by Mrs. Macgill, of North Esk Manse. Miss Murray has also been presented with a valuable carriage clock by the ladies of her committee, and a handsome umbrella by Miss Milne Woodside.

Poplar and Stepney Sick Asylum.—Last week an "at home" was given by the nurses of the Poplar and Stepney Sick Asylum, to bid farewell to Nurse Maud Frenche, who is leaving to be married and to live in Natal. During the evening the matron, in a few well-chosen and appropriate words, presented Nurse Frenche with a set of silver tea-spoons and sugar tongs on behalf of the medical and nursing staff, as a token of their affection and good wishes for her future happiness.

Nuneaton and District Cottage Hospital.—A pleasing ceremony took place at the Nuneaton Hospital on Thursday, last week, when the "new Victoria wing," which was built as a memorial of the Queen's Jubilee, was opened by Mr. Frank Newdigate, M.P., accompanied by his wife. On her arrival Mrs. Newdigate was presented by Miss Esme Johnson, grand-daughter to Mr. J. F. Johnson, J.P., of Attleboro Hall, with a lovely bouquet. After the opening ceremony Miss Macgowan, the popular matron, who was trained at St. Bartholomew's Hospital, London, was presented by the chairman, Mr, R. Stanley, with a purse containing £25, which had been voted to her by the committee and subscribers in recognition of the valuable services she has rendered during the three years she has held her present post. At the same time Mr. Guy Johnson handed her a superb basket of choice flowers, which had been subscribed for by the medical staff and friends.

### Appointments.

Addenbrooke's Hospital, Cambridge.—Miss Louisa Partridge has been appointed assistant matron. She was previously sister of the accident ward at the Seamen's Hospital, Greenwich.

Cottage Hospital, Clun, Salop.—Miss Helena Winney has been appointed matron. She was trained at the Leeds General Infirmary, and was previously for two years at the Cottage Hospital, Wallasey, Cheshire. She has since been charge nurse of the male wards at the R. N. Sea Bathing Infirmary, Scarborough.

The Infirmary, Isleworth.—Miss C. de la Fontaine has been appointed night superintendent. She was trained for three years at the Royal Infirmary, Glasgow, where she has since held the post of ward sister for two and a-half years. She possesses the midwifery certificate of the Rotunda Hospital, Dublin, and of the L.O.S.

Hospital for Diseases of the Nervous System, Belfast.—Miss Janet Elliot has been appointed lady superintendent. She was trained at the Paisley General Infirmary and Fever Hospital, and has since been nurse at Sunderland Infirmary, private nurse at Glasgow, nurse at the National Hospital for the Paralysed and Epileptics, London —where she received her certificate for massage—and district nurse on the Castle Huntley Estate, Perthshire.

Victoria Infirmary, Glasgow.—Miss Jessie Campbell has been appointed assistant matron; Miss Mary Coats matron of the Convalescent Home, Largs, Ayrshire; and Miss Isabel Duncan, night superintendent. Miss Campbell was trained at the Victoria Infirmary, and has since been matron of the Convalescent Home at Largs for three and a half years. Miss Coats was trained at the Victoria Infirmary and at the Maternity Hospital, Glasgow; and for the last three and a half years she has been Queen's Nurse at Tarbert, Loch Fyne. Miss Duncan was trained at the Royal Infirmary, and has since been sister-in-charge of wards at the Victoria Infirmary.

### Minor Appointments.

Margate Cottage Hospital.—Miss Maud Lane has been appointed staff nurse. She was trained at the Royal Albert Edward Infirmary, Wickham.

Addenbrooke's Hospital, Cambridge. — Miss Beatrice Brewin has been appointed night superintendent. She was trained at the Seamen's Hospital, Greenwich, and at the Hospital for Women, Soho.

City Hospital, Coventry.—Miss Ethel Worseldine has been appointed charge nurse. She was trained at St. Saviour's Infirmary, and has since been charge nurse at the Brook Hospital and at the Fever Hospital, Lower Tooting.

St. Leonard's Infirmary, Shoreditch.—Miss Alice Maud Mary Blumenthal has been appointed ward sister. She was trained at Highgate Infirmary, and has since been superintendent nurse at the City of London Hospital, Barnhill Hospital, Glasgow, and sister at Broughton Hospital.

Children's Hospital, Nottingham.—Miss Mabel Fox has been appointed sister. She was trained at the Clinical Hospital for Women and Children, Manchester, and was staff nurse there for two years. She has since been charge nurse in the women's wards at the Royal Northern Sea Bathing Infirmary, Scarborough.

Northern Convalescent Fever Hospital, Winchmore Hill.—Miss Hannah Maria Pudfield and Miss Alice Barber have been appointed charge nurses. Miss Pudfield was trained at Poplar and Stepney Sick Asylum, and Miss Barber at Fir Vale Infirmary, Sheffield. The latter has since been sister at the Mission Infirmary, Aston, and has also been engaged in district work at Chorlton-cum-Hardy.

### Wants and Workers.

Will anyone help a district nurse in getting a few toys for the children of the poor, for Christmas time. Old clothes will also be thankfully received by the nurse, Nurses' Home, Market Place, Atherton, Manchester.

### To Nurses.

We invite contributions from any of our readers, and shall be glad to pay for "Notes on News from the Nursing World," or for articles describing nursing experiences, or dealing with any nursing question from an original point of view. The minimum payment for contributions is 5s., but we welcome interesting contributions of a column, or a page, in length. It may be added that notices of entertainments, presentations, and deaths are not paid for, but, of course, we are always glad to receive them. All rejected manuscripts are returned in due course, and all payments for manuscripts used are made as early as possible at the beginning of each quarter.

### Everybody's Opinion.

### A SUPERINTENDENT NURSE APPEALS TO THE LOCAL GOVERNMENT BOARD.

"Poor Law Guardian" writes: Since the appearance of your note anent the East Preston Guardians and their superintendent nurse (Miss M. L. Rogers) I have obtained the local papers and read up the case. Whilst not attempting to decide between the Board and the nurse, it is apparent, on the face of the reports, that the Board has acted most unjustly in asking for the lady's resignation without even deigning to supply her with a copy of the Nursing Committee's Report, or to inform her in any way what they think they have to complain of. One writer, in a letter to the local Press, says there are "several justices (save the mark!) upon the Board: not one of them appears to be sufficiently versed in the first principles of English law, viz., that the accused should not only have the right to know the charge against them, but to hear the evidence and plead." With this I thoroughly agree. There is, however, one deduction to be made from this case, which I know is not a solitary one, and that is, the Local Government Board must amend its orders relative to the position of superintendent nurses. They have insisted, and rightly so, I fully believe, that these officers shall be appointed, and further that they must be properly qualified for the office. They have further properly decided that, so far as the nursing is concerned, the superintendent shall only be responsible to the medical nfficer. But these wise and beneficent orders are to a large extent rendered nugatory, and the life of the superintendent made one of incessant worry by the masters and matrons of workhouses, who almost without exception resent the appointment of superintendents of nurses, doubtless because they think it interferes with their autocratic power. I know cases where these officers give out as little clothing and bedding as they possibly can, take care that the proper nursing is interfered with as much as possible by sending clean linen, wearing apparel, medical requirements, nourishments, &c., as late in the day or week as they dare, causing the superintendent nurse to have to send time after time for them. This also has the effect of worrying the other nurses, who are taught to put the blame on the superintendent. In more than one case the nurses are encouraged to be as insubordinate as possible to the superintendent, knowing that the Board looks through the master's eyes and votes the superintendent and her office a nuisance. Already the Local Government Board have, at the request of three (or more) Boards, sanctioned the placing of the infirmary altogether under the control of the medical officer and superintendent nurse. When the unenlightened Boards have been compelled to do what the enlightened ones have asked for, and not till then, will the superintendent nurse (who is a professional person) be placed in her right position, her life at least be made bearable, and, what is perhaps even more important, the sick poor cease to be the shuttlecock between the professional officers (medical officer and superintendent) and the ofttimes ignorant and autocratic master and matron.

### AN OBJECT LESSON FROM CROYDON.

"The Matron of St. Mary's Hospital, Tenbury," writes: I fully agree with every statement in the very sensible paper, written by a "Matron of Experience," headed "An Object Lesson from Croydon." I have been matron of a small hospital for four years, and I am sorry to say I have had only too much experience with the class of nurse she mentions, and most aptly terms "untrustworthy." There are sadly too few women, either well-born or lowly, who go in for nursing from a real desire to help and comfort their suffering fellow creatures; too many who go in for it, because perhaps of a disappointing flirtation, or because they imagine they will look well in the uniform, and who do not care one atom for the welfare of their patients if they themselves can (by neglecting their duties) have a fairly easy time. I think there is nothing more discouraging to a matron, who has her patients' interests at heart, to find that her probationer, who is the only nurse she has, is one of these "untrustworthy" women. If, for instance, the matron has been kept indoors for a week or more, because her nurse is not trustworthy enough to be left with bad cases, then when there a lull in her work, and her patients are convalescent, she feels at liberty to accept an invitation to go out for the afternoon. She returns and goes round the wards, and finds that the medicines have been forgotten, temperatures have not been charted, the crumbs have not been swept out of the beds, the backs of bed-ridden patients not attended to, and hot-water bottles filled with lukewarm water. She is told, perhaps, that a doctor has been in to see his patients, and next time she sees the doctor he congratulates her for having been so fortunate as to obtain such a "very nice girl as probationer," and says that he is sure she will be "quite a treasure, so bright and intelligent." Alas! the matron knows well that the brightness is only put on for the occasion, for she has seen the different expression worn on the face of that nurse when she is viewed only by matron and patients. She knows it is no unusual thing to see the nurse go into the wards in the morning to do her work with a face as "long as a fiddle" and without uttering a word to the patients. No interest whatever is taken in them, and if a poor creature says that she has had a bad night, no word of sympathy is given. When the matron wishes to keep the poor sleepless one as quiet as possible in the afternoon, hoping that she will secure a little much-needed rest, the nurse talks and laughs loudly with the convalescent patients. I think that if the suggestion made by "Matron of Experience" could be carried out, we should hear less about unsatisfactory trained nurses. Of course many who pass now would not do so then, but we could do quite well without the "paragons" whom a "Matron of Experience" mentions. They would never pass through the matron's examination. I do not know Miss Julian, but I admire her courage in refusing to give certificates to nurses who did not deserve them. It would have been easier and pleasanter to have given them, although, if she had done so, she would have acted very wrongly. A matron's task is a hard one if she does her duty faithfully to her patients, her hospital, and her nurses, especially if she likes to be popular (and who does not?). She finds that there are often times when it is impossible to be both just and popular, and then there is great danger of her sacrificing justice to popularity, and rather than bear the brunt of being intensely disliked and unkindly spoken of by an untrustworthy nurse whom, in justice to hospital and patients, she ought to send away, she will be apt to overlook her faults, and bear with endless worries and disappointments. Finally, she will give the nurse a testimonial for one or two things that she does fairly well, not mentioning her faults, and will console herself with the thought that she has got comfortably quit of her. I hope that there are not many matrons who would act thus, and endanger the lives of future patients by allowing such a woman to go on with her profession. But there are some, and it seems to me that it would be wise to encourage Miss Julian in her act of moral courage in thus sacrificing her own popularity for the sake, not only of the Croydon Infirmary and its patients, but also for the sake of other hospitals and other patients whose lives may in the future be in the hands of these "untrustworthy women." I feel sure that Miss Julian will have the entire sympathy of every matron, sister, and nurse whose aim in life is to see the sick and dying in our hospitals kindly and properly treated.

Mr. W. Berry, F.R.C.S.I., of Wigan, writes: I have watched with much interest the various phases of the Croydon difficulty, where the matron refuses to sign certificates for nurses who have finished their training, and also the Oldham difficulty, where the medical officer objects to sign the certificates of training. I have read carefully the notes under the above heading by "A Matron of Experience," and would like to remark that these would have had much more weight if the name had been given. However, the most important point in each of these difficulties is what injustice is being done to the nurses, whom, I presume, have undergone the regular and full course of training. Now, no one regrets more than I do that a matron should be reduced to the position of an upper nurse and general housekeeper. But I take exception to the statement "that there is a decided tendency towards degeneration in the nursing profession at the present time." I have been interested in matrons and nurses for the last twenty-eight years, and can say from experience that the nurses of to-day are superior, both educationally and morally, to what they were at the commencement of my time, and that the raining its superior and I know that the present nurse is not any more wanting " in conscientiousness and other attributes of character," if we may fairly gauge the same by percentages, than her predecessor. The "untrustworthy nurse," as described by the matron, is not the rule. Such nurses are exceptions, and it is wrong to blame the majority for the faults of the minority; but how are we to get rid of this would-be nurse? By letting her go through her course of training and then refusing to sign her certificate, or refusing to give her a reference when she seeks another appointment? No. These women should be weeded out in their first month of trial; if one month is too short, then three months, or if you say no, well, then one year. Surely that is long enough for any matron to find out the faults and defects of a probationer; but to permit her to go on to the end of her training and refuse what she has earned is not just. Matron says: "At last a nurse of this sort scrapes through her examination and comes to the end of her three years' period of bondage, and now she expects to receive the certificate which will guarantee her as a properly-qualified nurse fit to hold responsible posts. She has never done anything sufficiently morally wrong, perhaps, to warrant her summary dismissal from the hospital, and yet she is utterly unfit to have the care of the sick and the issues of life and death in her hands. The question, therefore, stands thus: Is the public to have these three years' trained frauds fostered upon them or, by withholding certificates, are matrons to have the power of putting a stop to a crying evil of which they alone can be the proper judges?" Now this is a large order, and I wish to answer it in detail. First, if a nurse conducts herself so as to go through a three years' training and obtain sufficient knowledge, or knowledge equal to her fellow-nurses', she should have her certificate and is entitled to it. If she does anything morally wrong or otherwise to forfeit it she should have been prevented from going on with her training long before these years were over. We must remember that in her third year "she has been fit to have the care of the sick and the issues of life and death in her hands." Secondly, no individual matron should be allowed to be such an autocrat as to have the withholding of a certificate to one she has allowed to go through her training. A good matron should be able to discover the defects of a probationer long before her probation is over. What would be thought of a mistress who kept a cook for three years and then said she could not cook, and was unfit to do so? Or what would be thought of a medical man who kept an assistant in his practice for three years and then said he was unfit for his work? There are, I know, nurses and nurses, but matrons ought to remember that the nurses under them are not so smart as themselves. If they were so, we should have them all trying to be matrons. It is not a matter of honours in nursing, nor is a certificate of training a first-class testimonial. A doctor's diploma does not say he is as proficient, or that he ever will be, as his teachers; nor does it say he is as clever as his neighbour; all it does is to show that he has had the recognised training and profited thereby, so much that he was worthy of his certificate, and he is "allowed to have the issues of life and death in his hands." When a matron says, as a matron and a woman, "I would rather have a probationer of three months' standing to nurse me, had the probationer the essential qualifications, combined with brains and common-sense, than a nurse of six years' experience, &c., and who did not possess them," surely she is going back to the days of "Nurse Gamp," whose only recommendation was her brains in the shape of coolness and common-sense. A college of nurses won't do, and would not remedy the matter; what is required is that the "weeding out" process should be adopted at the commencement of the training and not at the end. I take it that the majority take to nursing as a profession, as a mode of earning a livelihood, and if they spend three years in trying to learn what they cannot, or are unfit for their duties afterwards, then a gross injustice has been done by allowing them to go on, and afterwards casting them to the winds. Matrons should remember this and also try to put themselves in the position of those so treated. How would they like it if they had been less fortunate, or how would they like their sisters treated in that way?

### How to Make a Travelling Tea-basket.

By the Travel Editor.

I have received so many letters requesting information on this thrilling question that it seems necessary to write a little account of the treasured article, for I can hardly give the necessary information in the correspondence column. The baskets such as I described are only to be bought at a wine merchant's; they hold six small bottles of champagne, are oblong and very light, close with a stick, and have a stout handle. It would be necessary to buy the champagne, too, for wine merchants would naturally not make a trade of their baskets, but sometimes they will oblige an old customer. In case you know no such obliging person and do not wish to invest in champagne, buy one of those brown baskets with slightly domed lids that artisans and railway men use to carry their dinners in. They are not quite so admirable as the champagne basket because the domed lid does not fit so well among other parcels in a railway rack. The dimensions I recommend are 15½ inches long by 9½ wide and 9½ deep. It must not be smaller, but an inch or so bigger would not signify. Buy two plates and two cups and saucers of enamelled tin—cost from 4d. to 6d. each—two knives, two forks, and two spoons. Then take broad black cotton elastic and sew it firmly round the sides of the basket inside in divisions, so as to hold the plates and saucers; sew similar divisions, but small, on the inside of the lid, to fix the knives, forks, and spoons. The cups must be placed in the body of the basket. Then buy a small travelling kettle and teapot, combined with a spirit lamp, of the kind that can be carried full of water—which means that they possess a screw lid and screw top to the spout. (Remember, in boiling, to remove the spout screw, or you will have a dreadful explosion.) These kettles can be bought almost anywhere, but certainly at Drews', Piccadilly Circus. Take a small common cocoa tin to hold your tea air-tight, and another for sugar, if you use it. I take with me a good store of grease-proof paper to wrap provisions in, because tins are too cornery and knobby to agree with the limited dimensions of a tea basket. Milk may be carried in an ordinary medicine bottle, but always start with the bottle quite full, or you will find that the jolting of the train has turned it into butter. Be careful not to forget matches; I have an ingenious pair of wires fixed into a Bryant and May's tin holder, which I clamp on to the lid, thereby avoiding the imminent danger of leaving the precious matches littering on the railway cushions.

### TRAVEL NOTES AND QUERIES.

Rules.

The competition is open to all. Answers must not exceed 500 words, and be written on one side of the paper only. The pseudonym, as well as the proper name and address, must be written on the *same* paper, and *not on a separate sheet*. Papers may be sent in for fifteen days only from the day of the publication of the question. All illustrations strictly prohibited. Failure to comply with these rules will disqualify the candidate for competition. Prizes will be awarded for the two best answers. Papers to be sent to "The Editor," with "Examination" written on the left-hand corner of the envelope.

N.B.—The decision of the examiners is final, and no correspondence on the subject can be entertained.

In addition to two prizes honourable mention cards will be awarded to those who have sent in exceptionally good papers.

How to Make a Tea Basket (Tea Drinker).—The needed information is too long for this column; I hope to put in a short account of the travelling tea basket this week. Thanks for your kind remarks. Appreciation is always very pleasant.

From Marseilles to Paris (March).—See answer to Tea Drinker concerning tea basket. There are two alternative routes from Marseilles to Paris. The first is *viâ* Lyons, and you could not be in a more beautiful centre than at Tarascon or Avignon: Nîmes, Tarascon, Arles, and Avignon, with Orange to the north, are all close together and delightful. Further north the route is not very interesting. The other way would be to strike north to Annecy, which is ideal. You pass Grenoble and Chambéry, but it is a pity to try to do too much. You must go up to Geneva to strike the Paris route again, which you join at Macon. You might ask Cook if he could give you tickets: they are not always able to do so on cross lines. It is no economy, but it is sometimes a convenience if you are inexperienced travellers.

Travelling Bath (March).—You can get the indiarubber travelling baths at any outfitter's. I got mine at the Army and Navy Stores. It is better to get them at a general outfitter's or at the Stores rather than at a macintosh shop, because they are decidedly cheaper. They vary in size, but one at 10s. 6d. is quite large enough for travelling and folds up into the size of an ordinary book. I found a small kettle full of boiling water, such as I could manage with my tea equipage, heated the water in my bedroom jug sufficiently, and so necessitated no extra demands for hot water, a rare luxury in foreign hotels.

### For Reading to the Sick.

If there had been any better thing, or more profitable to man's salvation than suffering, surely Christ would have showed it by word and example.—*Thomas à Kempis.*

Lord, give me grace to love Thee in my pain;
Through all disappointment, love Thee still;
Thy love my strong foundation and my hill,
Though I be such as cometh not again,
A fading spark upon the wane;
So evermore do Thou Thy perfect will,
Belov'd through all my good, through all mine ill,
Belov'd though all my love beside in vain—
If thus I love Thee, how wilt Thou love me,
Thou who art greater than my heart? (Amen!)
Wilt Thou bestow a part? Withhold a part?
The longing of my heart cries out to Thee—
The hungering, thirsting longing of my heart:
What I forewent wilt Thou not grant me then?

*Christina Rosetti.*

Thrice bless'd are they who feel their loneliness;
To whom nor voice of friends nor pleasant scene
Brings aught on which the sadden'd heart can lean.
Yea, the rich earth, garb'd in her daintiest dress
Of light and joy, doth but the more oppress,
Claiming responsive smiles and rapture high—
Till, sick at heart, beyond the veil they fly,
Seeking His Presence Who alone can bless.

*Newman.*

This "day of trouble" did not come to me without God; I am laid aside by illness, because so it pleased Him. O my God and Father, I am laid on this bed by Thy hand; Thou Thyself didst place me here. Thou hast given me much good health, and many comforts of life; and many, many of my days have been days full of outward blessings; but now it has pleased Thee to change Thy dealing with me, and to send me trouble. I humbly bow beneath Thy hand, I desire to "hear the rod, and Who hath appointed it."—*Micah* vi. 9.

And now, O my God, what dost Thou bid me especially do at this time? "Call upon Me in the day of trouble:" these are Thy words to me now. This is the time that is marked out by Thee; and this is what Thou biddest me do to-day, "Call upon Me!" I am not to wait till I am a little stronger, or till my trouble has in a measure passed away, or till someone has done me good and brought me relief; no, I am to wait for nothing; I am to call upon God "*in* the day of trouble," this very day.

Lord, I thank Thee that I may; I thank Thee that Thou dost invite me, and tell me to call upon Thee. And therefore I do call upon Thee. Now, from my bed, while no one is near, now, Lord, I call upon Thee. Not in my own name, not as being worthy to come to Thee or speak to Thee; but in the name of Jesus, my Saviour, my Advocate, in His name do I call upon Thee. Guard me against forgetfulness. I will put no trust in my own resolutions, I will trust in Thee alone. By Thy Holy Spirit fix in me the desire to glorify Thee in word and deed. Thou hast said, "I will deliver thee, and thou shalt glorify Me." Oh, give me the double blessing; give me deliverance from trouble, and give me a heart to praise Thy name, and evermore to live to Thee. Grant me to live as one whom Thou hast delivered, acknowledging Thee, cleaving to Thee, loving Thee, and serving Thee in a consistent godly life.

Thy life that has been dropped aside
Into Time's stream, may stir the tide
In rippled circles spreading wide.
The cry wrung from thy Spirit's pain
May echo on some far-off plain,
And guide a wanderer home again.
Fail—yet rejoice! because no less
The failure that makes thy distress
May teach another full success.—*A. Procter.*

### Notes and Queries.

**The Editor is always willing to answer in this column, without any fee, all reasonable questions, as soon as possible.**

**But the following rules must be carefully observed:—**

**1. Every communication must be accompanied by the name and address of the writer.**

**2. The question must always bear upon nursing, directly or indirectly.**

**If an answer is required by letter a fee of half-a-crown must be enclosed with the note containing the inquiry.**

*Cancer Patient.*

(97) Can you help me to find a comfortable home for a poor lady suffering from cancer?—*Miss C.*

She could not be better cared for than at the Cancer Hospital, Fulham Road, Brompton, S.W., or in the new cancer wings at the Middlesex Hospital, Mortimer Street, W. Apply to the respective secretaries for particulars. She would probably be admitted at the Friedenheim Hospital, Avenue Road, Swiss Cottage, or the National Free Home for the Dying, 82 The Chase, Clapham Common.

*Nursing Reserve.*

(98) 1. Can you tell me where to apply in order to have my name put on the Army Nursing Reserve? 2. Is the General Hospital, Tunbridge Wells, a good training school?—*Anxious.*

1. Apply the hon. secretary, the Army Nursing Reserve, 18 Victoria Street, Westminster. 2. It is a small hospital of 55 beds. Probationers are instructed by the resident medical officer and by the ward sisters; they receive a certificate upon satisfactorily completing a three years' course of training.

*Probationer.*

(99) Could you kindly tell me how to become a probationer in any hospital where a salary is given? I am a parlour-maid, strong and healthy.—*Edith D.*

Apply to the Matron of the Middlesex Hospital, Mortimer Street, W. The salary begins at £12 for the first year. If unsuitable, see the "Nursing Profession: How and Where to Train," which gives full particulars of terms of training at all the principal schools.

*A Year's Probationer.*

(100) Can you tell me of any hospital or home where a probationer would be taken for a year's training, and be paid a salary?—*A. W.*

A probationer's efficient training costs more than her work is worth during the first year. If you want training and salary at any of the large hospitals you must enter for three years. But you might apply to St. John's Hospital for Diseases of the Skin, Leicester Square, W.C., the North Lonsdale Hospital, Barrow-in-Furness, or the Kendal Memorial Hospital.

*Dispensing.*

(101) Could you tell me if there is any shorter course of instruction in dispensing than that of the Pharmaceutical Society—one suitable for a nurse who has some spare time during the winter?—*M. E.*

"The Certificate of Qualification to act as an Assistant in Compounding and Dispensing Medicines" of the Society of Apothecaries of London, Blackfriars, E.C., would probably take less time to gain, but it will not qualify you to act as an independent dispenser.

*House-fellow.*

(102) Do you think I could find a visiting nurse to share my rooms? I am a working gentlewoman, not very strong, and feel very lonely at night. Would it be of any use to advertise in THE HOSPITAL, and what would be the cost?—*A. P.*

Yes. Write to the Manager of THE HOSPITAL for terms for advertising.

*Massage.*

(103) 1. Would you kindly tell me the best place at which to learn, at a moderate fee, massage, with electricity, &c.? 2. How long would it take?—*W. H. C.*

1. The National Hospital for the Paralysed and Epileptic, Queen's Square, Bloomsbury, provides courses of excellent instruction. 2. Probably three months.

*Fuller Training.*

(104) Would you be so kind as to inform me how to gain admission as probationer to a hospital or workhouse infirmary; and if any objection is made to mental nurses for such an institution?—*Eliza M.*

Apply to the lady superintendents of nurses for information. You should have no difficulty in finding an institution willing to train you in general nursing. For lists of hospitals and infirmaries see the "Nursing Profession: How and Where to Train."

*Colonial Nursing Association.*

(105) 1. Is the Colonial Nursing Association under Government? 2. Is the remuneration, considering the incidental expenses, good? 3. Is the work good? Any help to obtain the above information will be gratefully received.—*An Unsettled Sister.*

1. It has the support of the Colonial Office. 2. Fair. 3. Yes. Apply for full particulars to Mrs. Pigott, The Colonial Nursing Association, Imperial Institute, W.

*Isolation Hospital.*

(106) Can you put me in the way of obtaining a complete list of all the Isolation Hospitals for Infectious Diseases in Great Britain?—*F. E. S.*

A full list appears on page 495 and following pages of "Burdett's Hospitals and Charities" for 1900.

**Standard Books of Reference.**

"The Nursing Profession: How and Where to Train." 2s. net; post free, 2s. 4d.
"The Nurses' Dictionary of Medical Terms." 2s.
"Burdett's Series of Nursing Text-Books." 1s. each.
"A Handbook for Nurses." (Illustrated.) 5s.
"Nursing: Its Theory and Practice." New Edition. 3s. 6d.
"Helps in Sickness and to Health." Fifteenth Thousand.
"The Physiological Feeding of Infants." 1s.
"The Physiological Nursery Chart." 1s.; post free, 1s. 3d.
"Hospital Expenditure: The Commissariat." 2s. 6d.

All these are published by THE SCIENTIFIC PRESS, Ltd., and may be obtained through any bookseller or direct from the publishers, 28 and 29 Southampton Street, London, W.C.

## Figures and Tables

**Figure f1:**